# Synthetic promoters went green: MinSyns bridge the gap between tunable expression and synthetic biology in plants

**DOI:** 10.1093/synbio/ysaa027

**Published:** 2020-11-27

**Authors:** Andrea Tagliani

**Affiliations:** Department of Biosciences, Università degli Studi di Milano, 20133 Milan, Italy

Precise control of gene expression is critical to allow the design of tunable synthetic gene circuits. To date, our ability to precisely predict orthogonal expression in plants lags well behind that of animals and bacteria. This is largely because traditional attempts to characterize plant promoters have found few reliable sequence patterns. Even the TATA box is found in a minority of plant promoters (1). As a result, plant biologists are still relying on a set of promoters that are not completely orthogonal and that often cannot ensure homogenous expression between different tissues; moreover, the length of these promoters, the local DNA environment of the insertion and other unknown factors often do not allow for tunable and specific expression.

Recently, where classical approaches aimed at characterizing a discrete set of *cis* elements in plant promoters have failed to provide a comprehensive answer, machine learning helped researchers to blaze a new path for synthetic promoter design. In a paper published in *Nucleic Acid Research*, Cai *et al.* (2) developed a set of minimal synthetic promoters (MinSyns) by mining from typical promoters used in plant biology a set of rules by which, through a computational approach, the researchers were able to build a set of small standardized *cis*-regulatory elements (CREs) exploitable for green synthetic biology.

The main fodder for the author’s machine learning approach consisted not of plant promoters, but of sequences derived from pathogenic plant viruses, which are widely used in plant biology to drive constitutive expression. They found that small CREs from a set of these promoters are regulated by an endogenous plant transcription factor. Indeed, deletion experiments confirmed the importance of these CREs in regulating expression.

The authors used the experimentally determined strength of these CREs to generate a quantitative score for each. They then developed a script, which randomly assembles minimal promoters composed of few CREs for which a score is assigned, based on the relative promoter strength. Using luciferase-based reporter assays, the authors first confirmed that their MinSyn library could be exploited to tune transient expression in plant protoplasts.

The authors then selected four MinSyns for further characterization in transgenic plants. MinSyn promoters predictably drove the constitutive expression of GUS or YFP in stable transgenic lines of *Arabidopsis thaliana*, *Brassica rapa* and *Nicotiana benthamiana* plants. Finally, the authors demonstrated that it is possible to build synthetic genetic circuits from MinSyns, allowing tunable expression of two genes and variable expression patterns depending on the number of cognate binding sites for an orthogonal TF.

The novelty of this work lies in the organisms themselves. Due to their capacity to produce secondary metabolites and photosynthetic abilities, plants are arguably the most suitable chassis for the production of drugs, sustainable foods, biofuels and bioplastics (3, 4). Yet, tunable expression in plants has remained elusive, due to poor characterization of plant promoters. This work has important implications for the potential to develop a set of reliable promoters with predictable outputs for plants.

*Conflict of interest statement.* None declared.
